# The impact of contour maps on estimating the risk of gastrointestinal stromal tumor recurrence: indications for adjuvant therapy: an analysis of the Kinki GIST registry

**DOI:** 10.1007/s10120-023-01444-8

**Published:** 2023-12-25

**Authors:** Ryugo Teranishi, Tsuyoshi Takahashi, Shinsuke Sato, Katsunobu Sakurai, Kentaro Kishi, Hisahiro Hosogi, Takuya Nakai, Yukinori Kurokawa, Junya Fujita, Toshirou Nishida, Seiichi Hirota, Toshimasa Tsujinaka

**Affiliations:** 1 Department of Gastroenterological Surgery, Osaka University Graduate School of Medicine, 2-2, Yamadaoka, Suita City, Osaka 565-0871 Japan; 2 Department of Gastroenterological Surgery, Shizuoka General Hospital, Shizuoka, Japan; 3 Department of Gastroenterological Surgery, Osaka City General Hospital, Osaka, Japan; 4 Department of Surgery, Osaka Police Hospital, Osaka, Japan; 5 Department of Surgery, Japanese Red Cross Osaka Hospital, Osaka, Japan; 6 Department of Surgery, Faculty of Medicine, Kindai University, Higashiosaka, Osaka Japan; 7 Department of Surgery, Yao Municipal Hospital, Osaka, Japan; 8 Department of Surgery, Japan Community Health Care Organization Osaka Hospital, Osaka, Japan; 9 Department of Surgical Pathology, Hyogo Medical University, Nishinomiya, Japan; 10 Department of Surgery, Izumi City General Hospital, Izumi, Japan

**Keywords:** Gastrointestinal stromal tumors, Stomach, Adjuvant therapy, Imatinib, Contour maps

## Abstract

**Introduction:**

Contour maps enable risk classification of GIST recurrence in individual patients within 10 postoperative years. Although contour maps have been referred to in Japanese guidelines, their usefulness and role in determining indications for adjuvant therapy is still unclear in Japanese patients. The aims of this study are to investigate the validity of contour maps in Japanese patients with GIST and explore the new strategy for adjuvant therapy.

**Materials and methods:**

A total of 1426 Japanese GIST patients who were registered to the registry by the Kinki GIST Study Group between 2003 and 2012 were analyzed. Patients who had R0 surgery without perioperative therapy were included in this study. The accuracy of contour maps was validated.

**Results:**

Overall, 994 patients have concluded this study. Using contour maps, we validated the patients. The 5-year recurrence-free survival rates of patients within the GIST classification groups of 0–10%, 10–20%, 20–40%, 40–60%, 60–80%, 80–90%, and 90–100% were 98.1%, 96.6%, 92.3%, 48.0%, 37.3%, 41.0% and 42.4%, respectively. We confirmed that this classification by contour maps was well reflected recurrence prediction. Further, in the high-risk group stratified by the modified National Institutes of Health consensus criteria (m-NIHC), the 10-year RFS rate was remarkably changed at a cutoff of 40% (0–40% group vs. 40–100% group: 88.7% vs. 50.3%, *p* < 0.001).

**Conclusion:**

Contour maps are effective in predicting individual recurrence rates. And it may be useful for the decision of individual strategy for high-risk patients combined with m-NIHC.

**Supplementary Information:**

The online version contains supplementary material available at 10.1007/s10120-023-01444-8.

## Introduction

Gastrointestinal stromal tumors (GISTs) represent the most common type of gastrointestinal mesenchymal tumors and are detected most often in the stomach, followed by the small intestine and other sites in the colon, esophagus, and peritoneal cavity [1–5]. Surgical resection with a negative margin remains the only therapeutic modality for cure.

Adjuvant therapy has recently been established for patients with a high-risk of recurrence GISTs [6, 7]. Three randomized phase III trials evaluating the efficacy of adjuvant therapy with imatinib mesylate (IM) demonstrated the efficacy of adjuvant therapy with IM for high-risk GIST recurrence after resection and revealed that recurrence-free survival (RFS) was significantly prolonged in patients treated with IM for a duration of 1–3 years, as compared with that in controls [7–10]. Nonetheless, the eligibility criteria for randomization of patients in each trial were different. The first trial (i.e., the American College of Surgeons Oncology Group Z9001 study) targeted patients with GIST measuring ≥ 3 cm in size and reported that adjuvant IM treatment for 1 year improved the RFS rate, as compared with that in controls. Following this study, patients in the high-risk group according to the National Institutes of Health consensus criteria (NIHC) and patients with tumor rupture were randomized in the Scandinavian Sarcoma Group XVIII/Arbeitsgemeinschaft Internistische Onkologie study [9]. The European Organisation for Research and Treatment of Cancer 62,024 study was the largest phase III trial that targeted patients with primary GIST who had high and intermediate risk based on the NIHC [8]. For these reasons, there is no consensus on the indications for adjuvant therapy. Therefore, predicting the patient-specific risk of GIST recurrence plays a crucial role in determining indications for adjuvant therapy.

Several risk-stratification systems for analyzing patients after radical resection of GISTs have been proposed. The NIHC was proposed in 2002 and have been widely used globally since then [11]. This classification is based on tumor size and the number of mitotic counts per 50 high-power fields (HPFs). However, further study showed that the location of GISTs was also one of the important and independent prognostic factors for recurrence in patients after radical resection. Miettinen et al. proposed the Armed Forces Institute of Pathology Criteria; this classification involved the location of GISTs in addition to the tumor size and number of mitotic counts [3]. The presence of tumor rupture, which is a strong adverse prognostic factor for GISTs and potentially important in determining patient prognosis [12–14], is absent in these classification systems. Therefore, Joensuu et al. proposed the modified NIHC (m-NIHC) [15], which considers ruptured GISTs as high-risk tumors irrespective of other features since tumor rupture is a clinically malignant factor for recurrence. Previously, we compared these three risk classifications and reported that m-NIHC exhibited the highest sensitivity for predicting recurrence; hence, we proposed it for the identification of candidates for adjuvant therapy [14]. Despite tumor size and mitotic count showing a nonlinear association with the risk of recurrence, these were estimated by linear modelling. Therefore, current methods of estimating individuals’ survival outcomes were faulty.

Joensuu et al. proposed contour maps, which enable risk classification of recurrence in individual patients. Contour maps consider tumor size, location, mitotic count, and rupture and consider the tumor size and mitotic count as continuous nonlinear variables [16]. They reported that contour maps were more detailed than other criteria with respect to the 10-year risk of GIST recurrence and were appropriate for estimating the individualized outcomes. However, although contour maps have been referred to in Japanese guidelines [17], their validity has not been confirmed in Asian patients. Therefore, the present study aimed to clarify the validity of contour maps in Japanese patients with GIST and explore the new strategy for adjuvant therapy.

## Methods

### Study design and patients

The GIST registry protocol was designed by the Kinki GIST Study Group. In this study, 1426 patients with GISTs diagnosed by a pathologist at each institution were retrospectively and prospectively enrolled from 39 hospitals between 2003 and 2012. In total, 737 patients were retrospectively enrolled between January 2003 and December 2007, and 689 patients were prospectively enrolled from January 2008. The eligible patients were required to have tumor morphology compatible with GIST and positive immunostaining for the KIT protein. Only tumors that were completely removed macroscopically during surgery were considered eligible. Patients with distant metastases at the time of primary therapy were excluded. In addition, since the pathological features of patients after neoadjuvant therapy might be altered, patients who received neoadjuvant therapy were also excluded from the study. Finally, since the adjuvant therapy has been reported to influence postoperative results, patients who received adjuvant therapy were also excluded from the study.

The Human Ethics Review Committee of Osaka University Graduate School of Medicine approved this study (No. 18424-2), and the institutional review board at each other participating institution approved the study protocol.

Most postoperative follow-ups were performed using contrast-enhanced computed tomography (CT) to detect recurrence and metastasis, per the Japanese guidelines for GISTs [17]. Data on patients’ prognoses, characteristics, and clinicopathological features, including age, sex, tumor location, size, mitotic count, and tumor rupture, were reviewed from medical reports. Maximum tumor size was measured using operative specimens. Data on histopathological features and the number of mitoses per 50 HPFs were obtained by examining specimens stained with hematoxylin and eosin. Mitoses were counted at the highest power, and the mean value was used for analysis after counting the fields twice.

### Evaluation methods for estimating GIST recurrence

The risk of recurrence in patients with GISTs was estimated using m-NIHC and contour maps [15, 16]. The m-NIHC divides the risk of recurrence into the following four categories: very low, low, intermediate, and high risk. Contour maps predict the GIST recurrence rate at 10 years after surgery and categorize GISTs into seven groups (0–10%, 10–20%, 20–40%, 40–60%, 60–80%, 80–90%, and 90–100%) according to tumor size, mitotic count (per 50 HPFs), location, and rupture [16]. Tumor size > 25 cm and mitotic count > 50 per 50 HPFs were assumed to be equivalent to 25 cm and 50 HPF, respectively.

### Statistical analysis

RFS was calculated from the date of surgery until the date of the first recurrence. Overall survival (OS) was calculated from the date of surgery until the date of death. The censored was the last date of survival confirmation. These were calculated for various subsets of prognostic factors using the Kaplan–Meier method and log-rank test. The χ^2^ test was used to estimate sensitivity. Two-sided *p* values of < 0.05 were considered as statistically significant. Statistical analyses were performed using JMP® Pro 16.0.0 (SAS Institute Inc., Cary, NC, USA).

## Results

### Patient characteristics

**Fig. 1:**
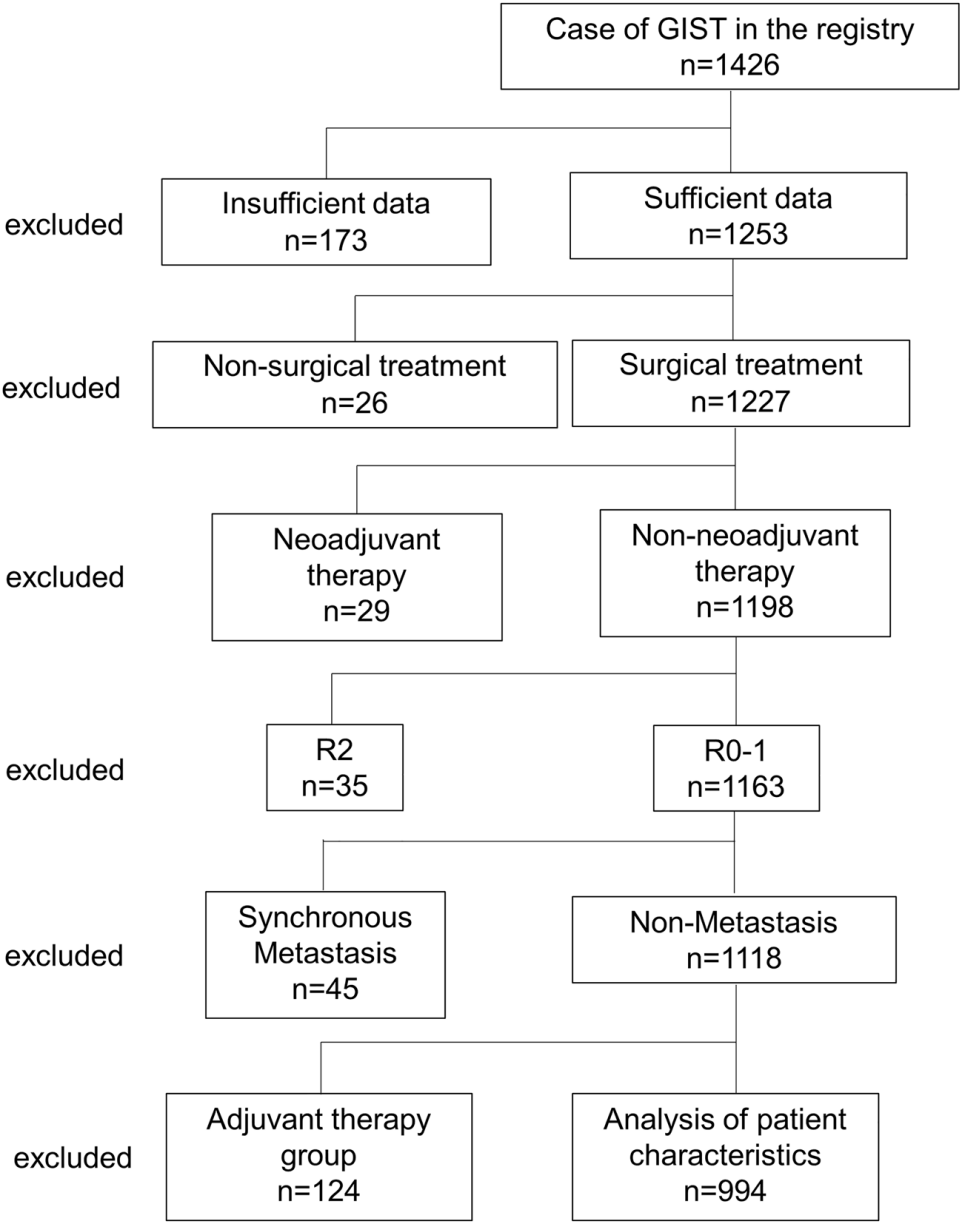
Flow chart of study recruitment

The study enrolled 1426 GIST patients from 39 hospitals. First, 173 patients with insufficient data were excluded. Furthermore, 26 patients who received non-surgical treatment and 29 patients who received neoadjuvant therapy were excluded. Next, 35 patients who underwent non-curative resection (R2) based on operative findings were excluded. There were 1163 patients who underwent curative resection (R0 and R1). Of these patients, 45 with synchronous metastases and 124 patients who received adjuvant therapy were excluded. Ultimately, 994 patients were included in this study (Fig. 1). The clinicopathological features of 994 patients are shown (Table 1). The median observation period was 6.1 years (range 0.02–20.3 years). Tumor locations included 779 GISTs in the stomach, 168 in the duodenum and small intestine, 29 in the large intestine, and 11 in the esophagus. The median tumor size was 3.3 cm (range 0.1–70 cm). The median number of mitoses per 50 HPFs was 2 (range 0–160). Tumor rupture was observed in 18 patients (1.8%). In total, 76 (7.6%) cases of recurrence were identified. Based on the m-NIHC, 183 patients (18.4%) were classified as having a very low risk, 456 (45.9%) as having a low risk, 144 (14.5%) as having an intermediate risk, and 211 (21.2%) as having a high risk. The recurrence rate for each group was as follows: 1.1% (2 patients) in the very low-risk group, 2.0% (9 patients) in the low-risk group, 4.9% (7 patients) in the intermediate-risk group, and 27.5% (58 patients) in the high-risk group. Based on the contour maps, 404 patients (40.6%) were classified as the 0–10% group, 297 patients (29.9%) as the 10–20% group, 164 patients (16.5%) as the 20–40% group, 51 patients (5.1%) as the 40–60% group, 23 patients (2.3%) as the 60–80% group, 17 patients (1.7%) as the 80–90% group, and 38 patients (3.8%) as the 90–100% group.

**Table 1:** Patients’ characteristics in non adjuvant chemotherapy group

	No. of patients (n = 994)	Median (range)
Age (years)		66 (18–92)
Sex (male: female)		
Male	512 (51.5)	
Female	472 (47.5)	
Unknown	10 (1.0)	
Primary site (n)		
Stomach (%)	779 (78.4)	
Duodenum and small intestine (%)	168 (16.9)	
Large intestine (%)	29 (2.9)	
Esophagus (%)	11 (1.1)	
Others (%)	7 (0.7)	
Tumor size (cm)		3.3 (0.1–70)
Mitotic counts/50HPFs		2 (0–160)
Tumor rupture		
Yes (%)	18 (1.8)	
No (%)	976 (98.2)	
Modified NIHC		
Very low (%)	183 (18.4)	
Low (%)	456 (45.9)	
Intermediate (%)	144 (14.5)	
High (%)	211 (21.2)	
Recurrence		
Yes (%)	76 (7.6)	
No (%)	918 (92.4)	
Observation period (years)		6.1 (0.02–20.3 years)

*HPFs* high power fields, *NIHC* The National Institutes of Health consensus criteria

### Risk-group stratification and outcome analysis for the m-NIHC and contour maps in patients

**Fig. 2:**
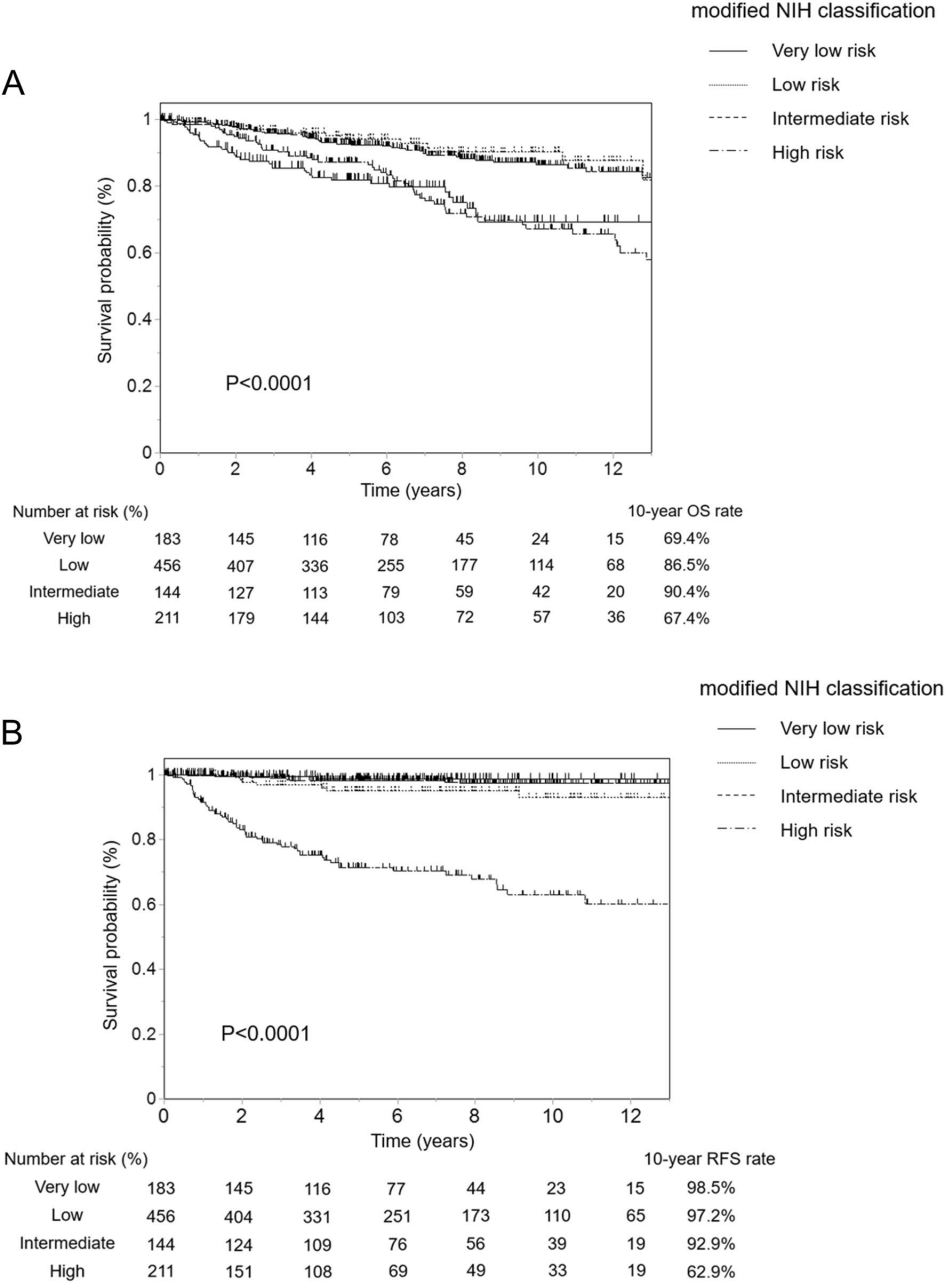
Prognoses of patients who did not receive adjuvant therapy according to the modified National Institutes of Health consensus criteria (m-NIHC). **A** Overall survival, **B** recurrence-free survival

**Fig. 3:**
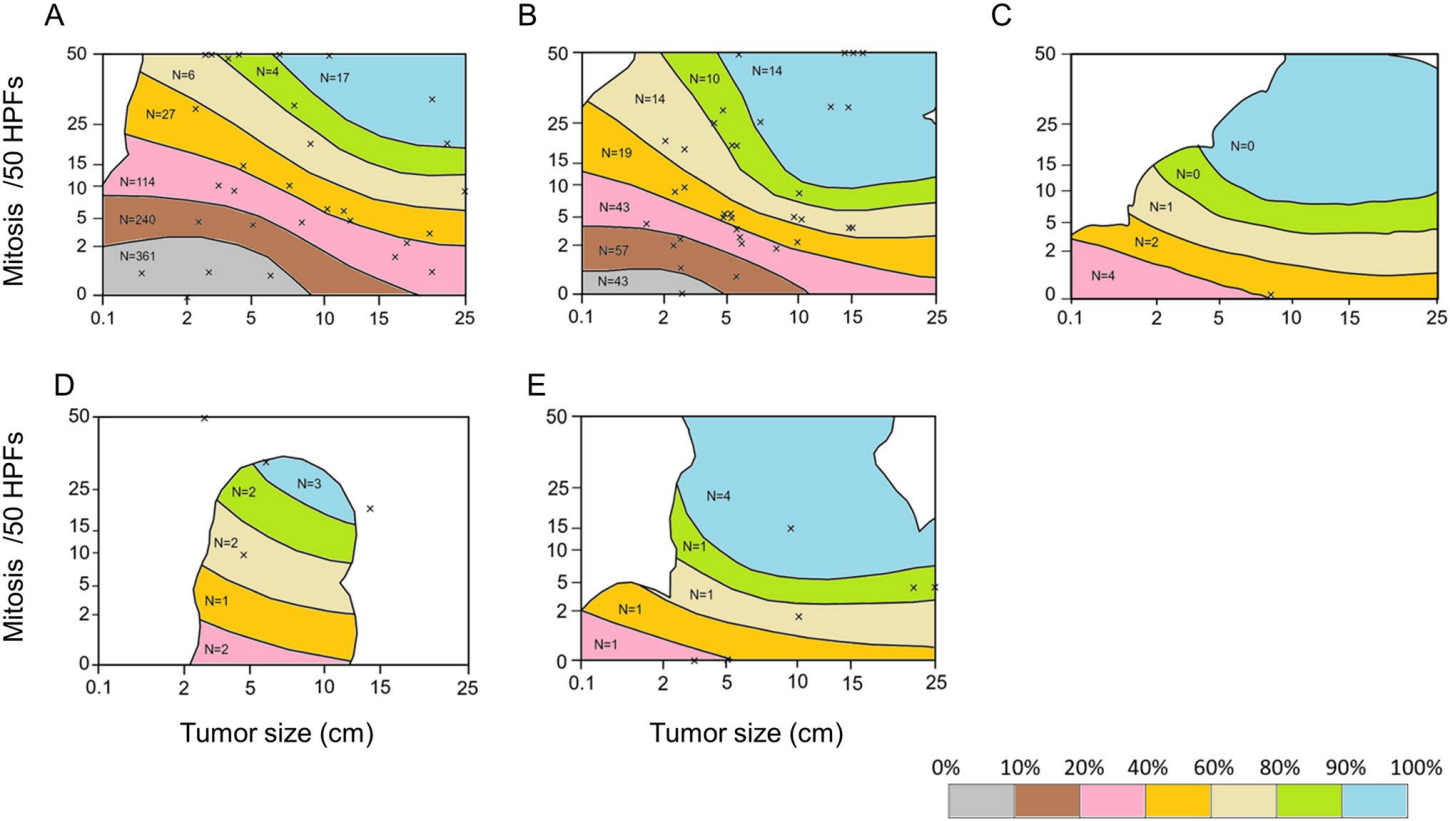
Recurrent cases plotted on contour maps. **A** Stomach gastrointestinal stromal tumors (GISTs) with no rupture, **B** non-stomach GISTs with no rupture, **C** extra-gastrointestinal stromal tumor with no rupture, **D** stomach GISTs with rupture, and **E** non-stomach GISTs with rupture. Tumor size over 25 cm and mitotic count over 50 per 50 high-power fields (HPFs) are assumed to be equivalent to 25 cm and 50 HPFs, respectively. This figure was referred from Joensuu et al. [16]

**Fig. 4:**
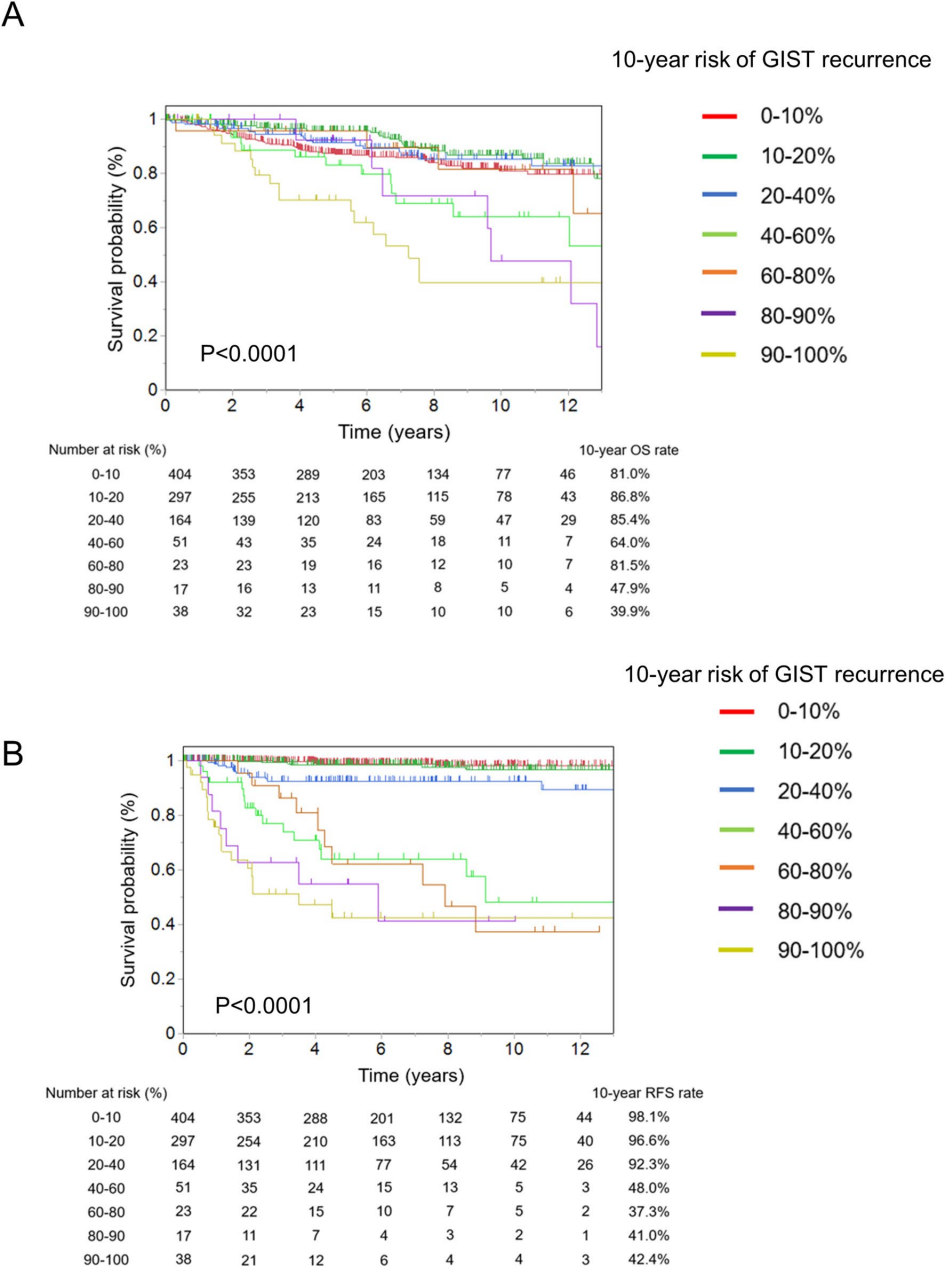
Evaluation of the prognosis of patients who did not receive adjuvant therapy using the contour maps. **A** Overall survival, **B** recurrence-free survival

We validated these 994 patients using the m-NIHC and contour maps. Recurrence occurred in 76 patients (7.6%). Based on the m-NIHC, 183 patients were classified as having a very low risk, 456 as having a low risk, 144 as having an intermediate risk, and 211 as having a high risk. The OS rate of the very low-risk group was similar to that of the high-risk group (Fig. 2A). The high-risk group showed significantly poorer prognosis (10-year RFS rate: 62.9%) than the other three risk groups (Fig. 2B). Next, we evaluated the risk of GIST recurrence using contour maps. Figure 3 shows only recurrent cases plotted into contour maps according to each tumor characteristic. There were 30 cases of recurrence in stomach GISTs without rupture, 4 in stomach GISTs with rupture, 35 in non-stomach GISTs without rupture, 6 in non-stomach GISTs with rupture, and 1 in extra-gastrointestinal GISTs without rupture (Fig. 3A–E). Evaluation of prognosis according to each risk classification using contour maps is shown in Fig. 4. The OS and RFS rates are shown in Fig. 4A, B. The 5-year RFS rates of patients within each of the GIST classification groups of 0–10%, 10–20%, 20–40%, 40–60%, 60–80%, 80–90%, and 90–100% were 98.1%, 96.6%, 92.3%, 48.0%, 37.3%, 41.0% and 42.4% respectively. We confirmed that the higher prediction group showed higher recurrence.

### Detailed analysis for high-risk classification based on the m-NIHC using the contour maps

**Fig. 5:**
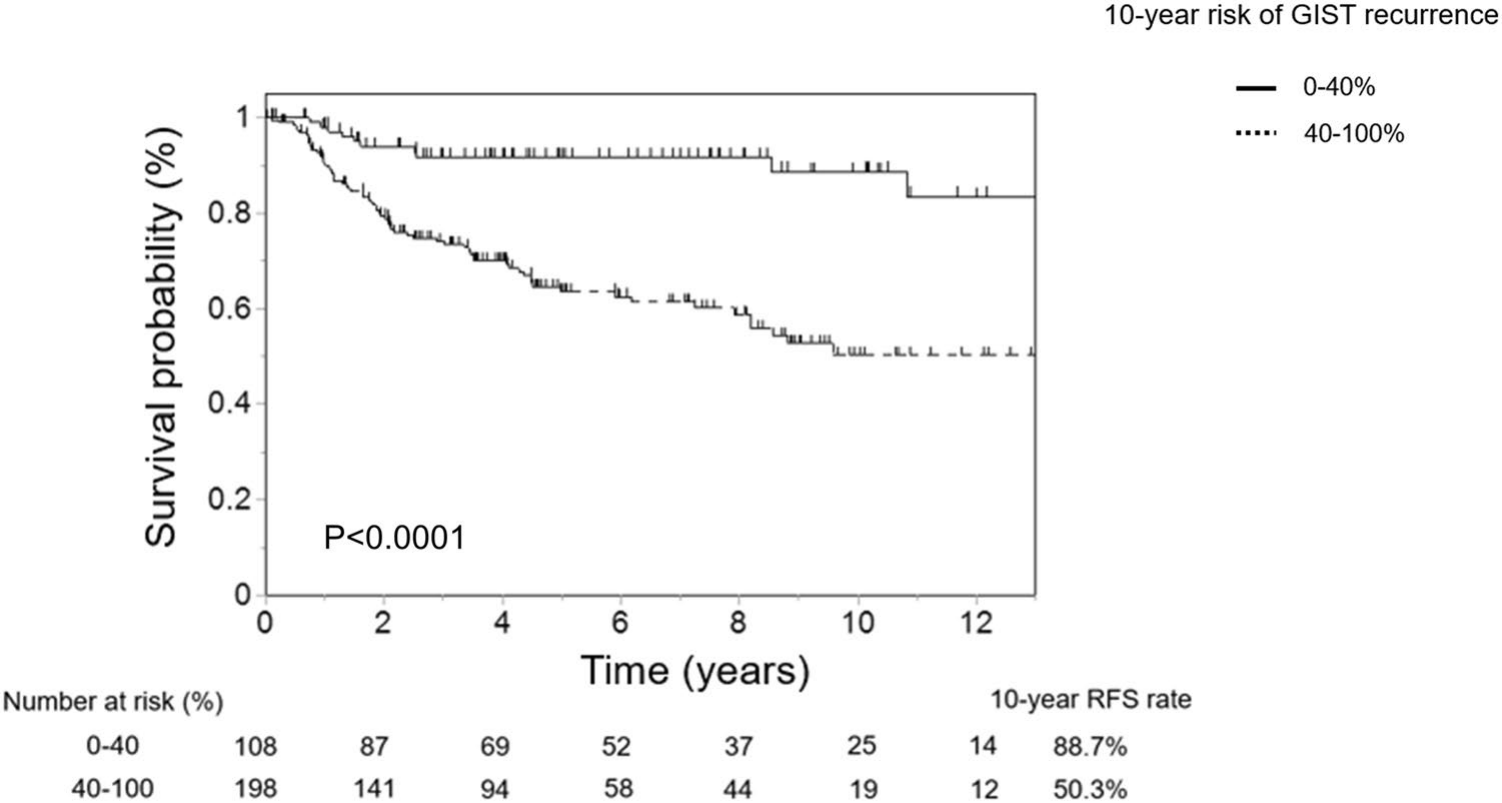
Evaluation of recurrence-free survival among patients in the high-risk group based on the m-NIHC with a cutoff value of 40% in the contour maps

Patients in the high-risk group based on the m-NIHC were reclassified using the contour maps (Fig. 5). In this population, a cutoff value of 40% also showed a significant difference in RFS, with the 10-year RFS rate being 88.7% in the 0–40% group and 50.3% in the 40–100% group (*p* < 0.001).

## Discussion

The Kinki GIST registry comprises the largest Japanese population in retrospective and prospective studies [14, 18–22]. Because this cohort study included consecutive patients in each hospital, this patient population reflects the characteristics of real-world Japanese patients with GIST. The proportion of patients with gastric GISTs was higher than that reported by previous international studies [12, 16]. In addition, there were fewer high-risk GISTs and more low-risk GISTs based on the m-NIHC, as compared with those in international reports [15, 16, 23]. This might be attributable to the well-established screening system for gastric cancer in Japan, which can detect several asymptomatic GISTs [14, 24]. Finding asymptomatic tumors using established screening systems improves prognosis. In fact, the overall prognosis for patients who did not receive adjuvant therapy in this study was better than that previously reported for both 10-year OS and RFS rates (80.1% and 89.6%, respectively) [16, 23, 25]. The 10-year RFS rate of the high-risk group was significantly lower than that of the other risk groups. This result suggests that the m-NIHC had a high sensitivity (76.3%) for predicting recurrence, and this criterion is clinically important in the selection of adjuvant therapy candidates, as previously described [26].

This study is the first to validate contour maps in the Japanese population. Recurrent cases are plotted in the contour maps, which estimate the gradient recurrence risk for each patient. First, we evaluated the patients’ recurrence risk according to the contour maps, and their prognoses were evaluated. The predicted recurrence in each patient aligned well with their prognoses, with rates of recurrence within 10 years increasing with the predicted risk of recurrence. Furthermore, the actual recurrence rate was lower than the estimated recurrence rate, suggesting that the recurrence rate in Japanese patients might have more optimistic prognoses than those in other populations.

Undergoing adjuvant IM for 3 years has been established as the standard treatment for patients with a high risk of relapse, based on randomized trials [9, 17, 27]. However, the benefit for RFS seemed to decrease after the end of adjuvant therapy, with an increasing risk of relapse during this period [7–9]. Thus, adjuvant therapy cannot eradicate micro-residual GISTs but may merely delay the time of recurrence. Nishida et al. reported that patients with potential micrometastases are recommended to take adjuvant therapy for more than 3 years [28]. Furthermore, some clinical trials are ongoing to determine whether extending adjuvant therapy with IM over 3 years can further reduce the risk of relapse and improve OS in patients with high-risk GISTs (NCT02413736, NCT02260505 and NCT01742299). However, it remains unclear for which high-risk patients are eligible for over 3 years. According to our results, the prognosis was different in the high-risk group according to m-NIHC. And, it may be useful for determination of the candidate for more intensive therapy. Thus, patients and surgeons will be able to obtain more detailed information from the contour maps with high sensitivity to recurrence (Online Resource 1) to make personalized decisions for postoperative treatment.

Tumor rupture is a serious risk factor for recurrence. Most ruptured GISTs are associated with recurrence at follow-up, and patients with ruptured GISTs have significantly shorter RFS than that those without rupture [22, 29, 30]. In the present study, the rate of tumor rupture was 1.8% in this population. The 10 year-RFS rate of the patients with tumor rupture was quite worse than that of high risk patients without tumor rupture (Online Resource 2). Therefore, it is considered reasonable to separate ruptured GIST patients and non-ruptured patients in the contour maps.

Neoadjuvant therapy with IM has been shown by both prospective and retrospective studies to effectively decrease tumor size, thereby facilitating ease of surgery and resulting in less morbid, organ-preserving operations [31–33]. For these patients, conventional risk classification is not applicable because pathological features are affected by IM. In particular, the mitotic counts of the specimen after neoadjuvant therapy have been reported to decrease remarkably, and we cannot fit patients into the m-NIHC or contour maps [34]. ^18^F-Fluorodeoxyglucose positron emission tomography/CT (^18^F-FDG PET/CT) is noninvasive and has been reported to be an effective imaging technique for assessment of malignancy and monitoring responses to IM therapy in GIST [34–39]. Proper selection of patients who require neoadjuvant therapy and risk evaluation after neoadjuvant therapy is important. However, prognostic factors such as mitotic count, exact tumor size, and tumor rupture during surgery can only be assessed postoperatively. In this regard, ^18^F-FDG PET/CT may aid in predicting the prognosis of neoadjuvant therapy patients. Further studies are needed to evaluate the possible role of ^18^F-FDG PET/CT in pathological assessment.

This study has some limitations. First, as this study was a registry study that included patients whose data were collected retrospectively, the follow-up period and way it was conducted were not standardized. Second, the very low-risk GISTs classification as defined by the m-NIHC included many incidental conditions of other serious diseases. In fact, while there were few cases of recurrence, 38 patients died during the observation period, and the 10-year OS rate was 69.4%, similar to that in the high-risk group (Online Resource 3). Of the patients who died in the very low-risk group, 92.1% were due to other diseases that were mainly the triggers for finding those small GISTs. Therefore, OS of the very low-risk GIST group does not reflect its own prognosis. Third, mutational analysis is known to be important in deciding whether to administer IM [7, 40–42]. However, most patients in this study did not undergo mutational analysis. Therefore, we could not evaluate the response to adjuvant therapy by genetic mutations. Additionally, since this study was excluded the high-risk patients with perioperative therapy, it might have some bias. However, this study was mainly based on patients admitted before 2012, when the standard treatment of 3-year adjuvant therapy was established, the ratio of patients with perioperative therapy was relatively low. Nevertheless, since the data were based on consecutive patients in each participating hospital, the study reflects real-world data on Japanese patients.

In conclusion, contour maps are effective in predicting GIST recurrence after primary surgery in the Japanese population. Contour maps may be useful in combination with the m-NIHC for the consideration of more individual indications for adjuvant therapy.

### Supplementary Information

Below is the link to the electronic supplementary material.

Supplementary file1 (TIF 791 kb)

Supplementary file2 (TIF 864 kb)

Supplementary file3 (PDF 72 kb)

## Data Availability

The data that support the findings of this study are available upon request from the corresponding author, Tsuyoshi Takahashi. The data are not publicly available because they contain information that can compromise the privacy of the research participants.
