# Supplementary figures and images for: The impact of contour maps on estimating the risk of gastrointestinal stromal tumor recurrence: indications for adjuvant therapy: an analysis of the Kinki GIST registry
Source: Gastric Cancer. 2023 Dec 25;27(2):355–65. doi: 10.1007/s10120-023-01444-8 (PMC10896809; doi:10.1007/s10120-023-01444-8)

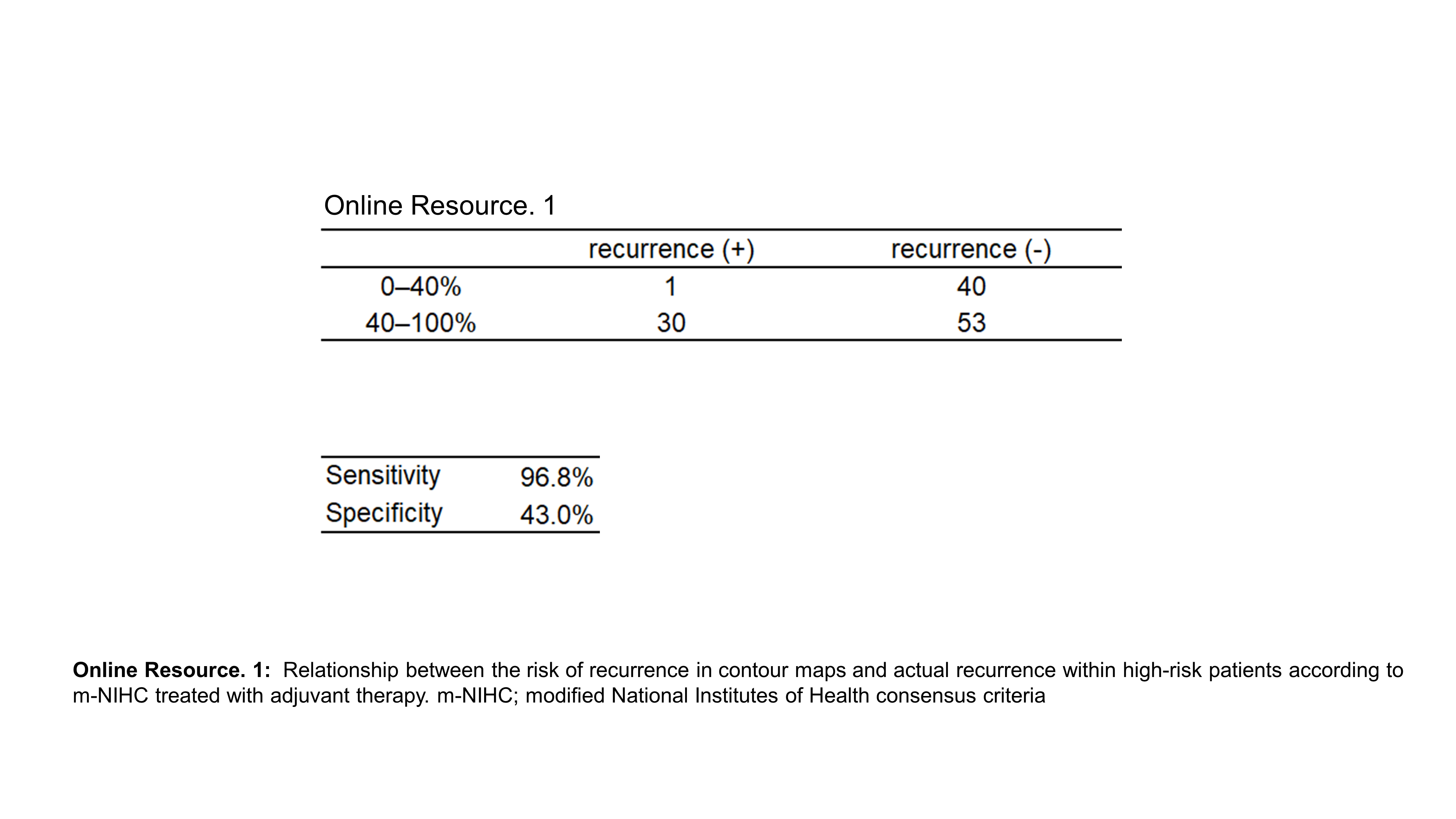

Supplement: Supplementary file 1 — Supplementary file1 (TIF 791 kb) [file 10120_2023_1444_MOESM1_ESM.tif]

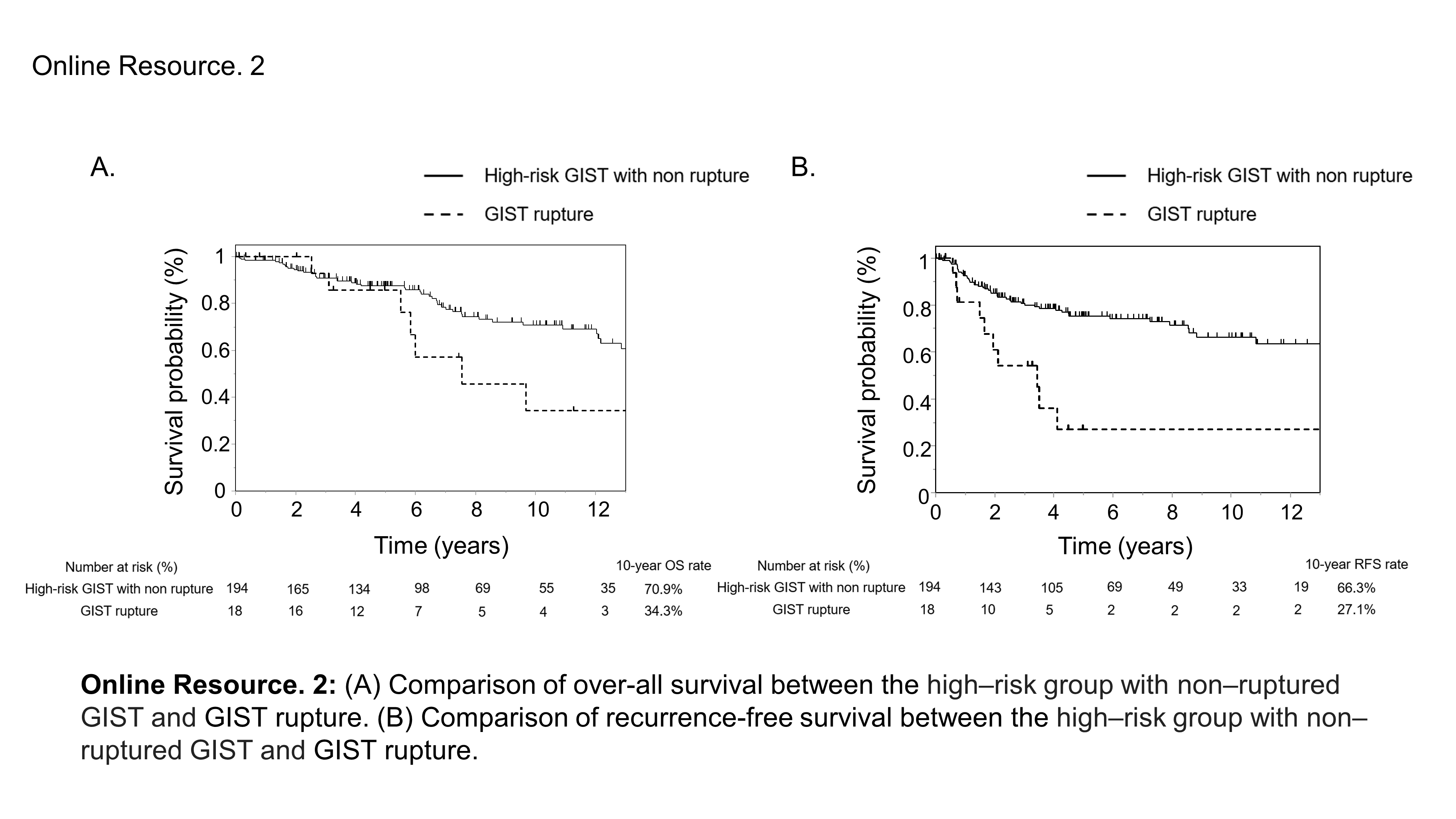

Supplement: Supplementary file 2 — Supplementary file2 (TIF 864 kb) [file 10120_2023_1444_MOESM2_ESM.tif]
