# Supplementary material for: The impact of contour maps on estimating the risk of gastrointestinal stromal tumor recurrence: indications for adjuvant therapy: an analysis of the Kinki GIST registry
Source: Gastric Cancer. 2023 Dec 25;27(2):355–65. doi: 10.1007/s10120-023-01444-8 (PMC10896809; doi:10.1007/s10120-023-01444-8)
Supplement: Supplementary file 3 — Supplementary file3 (PDF 72 kb) [file 10120_2023_1444_MOESM3_ESM.pdf]

Online resource. 3 The cause of death in each modified NIHC groups

Modified NIHC	Primary disease (N, %)	Other diseases (N, %)
Very low	3 (7.9%)	35 (92.1%)
Low	3 (6.7%)	42 (93.3%)
Intermediate	1 (8.3%)	11 (91.7%)
High	30 (60.0%)	20 (40.0%)

NIHC; The National Institutes of Health consensus criteria
